# A multi-stage computational pipeline for repurposing FDA-approved drugs: application to EGFR C797S–mutant NSCLC

**DOI:** 10.3389/fchem.2026.1753911

**Published:** 2026-03-31

**Authors:** Mansour S. Alturki, Reem A. Alkhodier, Abdulaziz H. Al Khzem, Ohood K. Almuzaini, Saad M. Wali, Khalid N. Aldosarry, Abdulaziz A. Halawi, Mohammed F. Aldawsari, Wajin R. Alruwili, Mohamed S. Gomaa

**Affiliations:** 1 Department of Pharmaceutical Chemistry, College of Pharmacy, Imam Abdulrahman Bin Faisal University, Dammam, Saudi Arabia; 2 Department of Pharmaceutical Sciences, College of Pharmacy, King Saud bin Abdulaziz University for Health Sciences, Riyadh, Saudi Arabia; 3 King Abdullah International Medical Research Center, Riyadh, Saudi Arabia; 4 Ministry of National Guard - Health Affairs, Riyadh, Saudi Arabia; 5 Department of Pharmacology and Toxicology, College of Pharmacy, Umm Al-Qura University, Makkah, Saudi Arabia; 6 College of Pharmacy, Imam Abdulrahman Bin Faisal University, Dammam, Saudi Arabia; 7 Department of Pharmaceutics, College of Pharmacy, Prince Sattam Bin Abdulaziz University, Al-kharj, Saudi Arabia

**Keywords:** covalent inhibitors, drug repurposing, EGFR C797S, molecular docking, molecular dynamics, NSCLC, virtual screening

## Abstract

**Background/Objectives:**

Non-small cell lung cancer (NSCLC) is a leading cause of cancer-related death globally. Resistance to third-generation epidermal growth factor receptor (EGFR) tyrosine kinase inhibitors, particularly due to the C797S mutation, poses a significant clinical challenge. This study utilized a comprehensive multiphase computational drug repurposing approach to discover FDA-approved medications that may be effective against the EGFR C797S mutation.

**Methods:**

A library of 1,650 compounds from the ZINC15 database was subjected to shape-based screening, followed by hierarchical molecular docking using high-throughput, standard precision, and extra-precision methods. The top candidates were further analyzed using MM-GBSA binding free-energy calculations, covalent docking simulations, and three 300-ns molecular dynamics simulations to evaluate the binding stability and interaction persistence under dynamic conditions.

**Results:**

Among the evaluated compounds, doripenem, norgestrel, oxymetholone, norethisterone, and ertapenem exhibited high docking scores and consistent interactions with crucial hinge-region residues, such as MET-793 and mutant SER-797, within the ATP-binding site. Through molecular dynamics analyses, ertapenem and oxymetholone were identified as the most stable complexes, exhibiting minimal root-mean-square deviation fluctuations and maintaining hydrogen-bond networks similar to that of the reference inhibitor osimertinib.

**Conclusion:**

These findings suggest that ertapenem and oxymetholone are promising structurally unique scaffolds for targeting osimertinib-resistant EGFR C797S–driven NSCLC. While experimental validation is necessary, this study provides a computational framework for swift drug repurposing and lays a rational foundation for future biochemical evaluation and structure-guided optimization of next-generation EGFR inhibitors.

## 1 Introduction

Lung cancer was the most frequently diagnosed malignancy in 2022. It is also the leading cause of death from malignant tumors in that year. The World Health Organization had stated that in 2020, lung cancer resulted in up to 1.8 million deaths out of the 10 million deaths worldwide (Sung et al., 2021). Among the various types of lung cancer, 80%–85% of lung cancer cases are non-small Cell Lung Carcinoma (NSCLC) (Duma et al., 2019). Epidermal growth factor receptor (EGFR) is a tyrosine kinase receptor that regulates cellular proliferation and growth. Its extracellular domain binds to a growth factor ligand, which causes the receptor to adopt an active conformation, dimerize, phosphorylate tyrosine residues, and induce biological effects through downstream signaling mechanisms (Wee and Wang, 2017). However, the receptor may induce carcinogenesis by altering downstream signaling when mutated by gain-of-function mutations; hence, EGFR mutagenesis may occur through overexpression, auto-activation, and chromosomal rearrangements (Rosell et al., 2009). EGFR mutations account for 49.1% and 12.8% of diagnosed cases of NSCLC in the Asian and European populations, respectively (Melosky et al., 2021). In addition to oncogene mutations, the protracted inflammatory signaling and remodeled immune micro-environment of diseased tissues are also recognized as playing an important role in modulating the pathway of cell survival, including pulmonary endothelium and parenchymal tissues (Wang et al., 2023; Wang et al., 2024). Prior to the discovery of targeted EGFR therapy, the estimated survival rate for patients with NSCLC undergoing chemotherapy was less than 1 year (Schiller et al., 2002). EGFR inhibitors have revolutionized oncology and shaped the history of NSCLC treatment (Mok et al., 2009). First- and second-generation tyrosine kinase inhibitors (TKIs), such as erlotinib and afatinib, respectively, show positive initial responses. However, cancer cells undergo specific mutations that reduce the efficacy of these drugs (Pluzanski et al., 2020). The most common mutations associated with acquired resistance are exon 19 deletion, exon 20 point mutation T790M, and exon 21 point mutation L858R (Yu et al., 2013). There is a 2-year decrease in mean survival associated with the T790M mutation, which is observed in 50%–60% of TKI-resistant cases (Chai et al., 2020). Drug resistance associated with such mutations, as well as contributing factors such as angiogenesis and metastasis, highlights the crucial need to consider new therapeutic alternatives. However, the development of new drugs entails substantial financial and time constraints.

TKIs prevent the phosphorylation of tyrosine residues in the ATP-binding pocket, thereby terminating the downstream cascades of action. The exchange of the threonine residue of exon 20 with methionine generates the mutation T790M. This larger methionine contributes to resistance against TKIs by creating steric hindrance at the gatekeeper site and increasing the affinity for nucleoside triphosphate, which causes receptor activation over TKI binding (Soucheray et al., 2015). Osimertinib is the first third-generation tyrosine kinase inhibitor (TKI) approved for treating T790M mutation-positive NSCLC. It irreversibly inhibits the phosphorylation process, thereby inhibiting cancer proliferation, growth, and survival (Cross et al., 2014). However, the predominant EGFR-dependent mutation causing resistance to osimertinib has emerged by changing the cysteine residue at 797 codon to serine (Thress et al., 2015). Fourth-generation EGFR-TKIs, such as BLU-945, BBT-176, TQB-3804, H002, and BDTX-1535, are being evaluated for their ability to target multiple EGFR mutations with improved selectivity and efficacy, including activity against triple-mutant variants and CNS metastases. However, these are still in the preclinical or early stages of clinical trials (Le et al., 2025; Leonetti et al., 2019).

Drug development takes more than 12 years and costs approximately $1.8 billion per drug. Consequently, contemporary drug discovery strategies may reduce both the expense and duration between development and accessibility as therapeutic alternatives (Shaker et al., 2021). Drug repurposing examines drugs that are already approved for use and have established pharmacokinetic, pharmacodynamic, and safety profiles (Kulkarni et al., 2023). In silico techniques, such as virtual screening, are increasingly used to expedite the initial stages of development and examine large databases (Chang et al., 2023). This computerized method has advantages in identifying early-stage drug candidates with the best chance of success in the screening process. Using this technique, we screened 1,650 FDA-approved drugs from the ZINC 15 database. Given the safety and efficacy of existing FDA-approved medications, our objective was to identify novel drug candidates from this database that share similar features.

The development of the EGFR C797S mutation after treatment with third-generation tyrosine kinase inhibitors, especially osimertinib, poses a significant challenge in the treatment of non-small cell lung cancer. Currently, there are no approved treatments that specifically target this resistant mutation, and fourth-generation EGFR inhibitors are mostly in the early stages of clinical trials. Considering the high cost and lengthy process of new drug discovery, repurposing existing drugs presents a practical and appealing alternative, utilizing compounds with known pharmacokinetic and safety profiles. However, there is a lack of systematic computational studies to identify FDA-approved drugs targeting the EGFR C797S mutant, which accounts for its altered covalent-binding properties and structural flexibility (Rangachari et al., 2019). This study aimed to create and implement a comprehensive, multi-layered *in silico* repurposing strategy to identify chemically diverse FDA-approved drugs that inhibit the EGFR C797S kinase domain. Instead of focusing solely on osimertinib-like structures, we aimed to investigate diverse chemotypes that could stabilize the ATP-binding pocket through sustained non-covalent interactions and engage SER-797 with electrophilic warheads or proximity-enabled covalent methods. To accomplish this, we combined shape-based screening, hierarchical molecular docking, induced-fit and covalent docking, MM-GBSA free-energy calculations, and extensive triplicate molecular dynamics simulations to refine the candidates while explicitly considering receptor flexibility and binding mode stability. Another goal was to use molecular dynamics simulations not only as a post-docking validation tool but also as a crucial filter to differentiate transient binders from ligands that can maintain stable interactions with key hinge-region residues under dynamic conditions. By comparing all candidates against the clinically used inhibitor, osimertinib, within a single computational framework, we aimed to establish objective prioritization criteria and gain mechanistic insights into how repurposed scaffolds might overcome the loss of irreversible Cys-797 binding. Ultimately, this study aimed to propose FDA-approved compounds for experimental testing against EGFR C797S–driven NSCLC and offer a transferable computational approach for addressing kinase resistance mutations more broadly.

## 2 Experimental

### 2.1 Software

The Schrödinger Maestro molecular modeling software (Version 2025-1) was used for the computational study (Bonczek et al., 2018; Patil et al., 2025; Rajagopal et al., 2017). The computational work was performed on a desktop workstation with a 13th Gen Intel(R) Core (TM) i9-13900 KF Processor, operating on the Ubuntu 22.04.5 LTS Operating System, and equipped with NVIDIA GeForce RTX 4090.

### 2.2 Database preparation

A library of FDA-approved medications was obtained from the ZINC15 online server (https://zinc.docking.org/), a publicly accessible database comprising over 750 million commercial chemicals. A total of 1,650 compounds were downloaded and stored in the 2D Structure-Data File (SDF) format and then imported into Maestro. A total of 1,650 compounds were prepared using the LigPrep tool in Maestro to incorporate missing hydrogen atoms, construct and energetically minimize outputs, optimize with the OPLS3e force field, convert to their corresponding 3D chemical structures, and ionize stereoisomers at a neutral pH of 7.0 ± 2.0 using the Epik ionizer. The generation of tautomers and de-salted ligands was permitted, maintaining a particular chirality for the stereoisomeric compounds, with each ligand capable of producing a maximum of 32 isomers (Bonczek et al., 2018; Kumar and Zhang, 2018).

This resulted in the creation of 2,792 total compounds. The initial 2,792 compounds were processed using the Filter Duplicates tool in Maestro to eliminate stereoisomeric duplicates, yielding 1,590 unique compounds. The Ligand Filtering method was used to select compounds that exclusively possess an electrophilic functional group, explicitly targeting the Michael acceptor capability to target SER-797 in EGFR. This yielded 102 final compounds for docking.

### 2.3 Crystal structures and protein preparation

High-resolution crystallographic data provide the most reliable structural basis for structure-based drug discovery targeting mutant kinases. In this study, the X-ray crystal structure of the EGFR kinase domain containing the C797S mutation (PDB ID: 6LUD) was chosen as the primary receptor model because it directly captures a clinically relevant resistance substitution responsible for the loss of covalent inhibition by third-generation tyrosine kinase inhibitors (Kashima et al., 2020). The structure was solved in complex with osimertinib and represents the ATP-bound state of the mutant receptor, thus providing an experimentally validated binding site geometry for benchmarking the accuracy of the docking and molecular dynamics simulations.

Prior to computational use, the 6LUD structure was scrutinized for the structural completeness of the activation loop, hinge region, αC-helix, and gatekeeper pocket, which are critical determinants of inhibitor recognition in resistant EGFR variants. Compared to wild-type EGFR structures and earlier T790M-only mutants, the C797S substitution alters the electrostatic environment of the catalytic cleft and removes the nucleophilic thiol required for irreversible covalent inhibition of EGFR. These features render C797S-containing structures mechanistically distinct, requiring tailored modeling strategies to study them. Thus, the availability of this particular mutant structure allowed for a direct investigation of the resistance mechanisms and alternative binding strategies.

The Protein Preparation Wizard tool in the Schrödinger Maestro software was used to prepare the crystal structure of EGFR C797S (PDB code: 6LUD) obtained from the Research Collaboratory for Structural Bioinformatics (RCSB) Protein Data Bank (PDB) (Kashima et al., 2020). Further steps were taken to eliminate unnecessary water molecules, add hydrophilic hydrogens, and set the pH to 7.4 using PROPKA. The Optimized Potentials for Liquid Simulation 3 model was used to optimize the attained structure. A root-mean-square deviation (RMSD) of 0.30 Å was used for non-hydrogen atoms. Energy minimization was performed using the OPLS3e (Harder et al., 2016).

### 2.4 Receptor grid generation

A receptor grid generation tool was used to generate a 20 × 20 × 20 Å box centered on the co-crystallized ligand of the target protein. A van der Waals (VDW) scaling factor of 1.0 and a partial charge cutoff of 0.25 for receptor atoms were selected (Ban et al., 2018).

### 2.5 Shape screening

The shape screening tool was used as a filter in Schrödinger Maestro software. We initiated the process by incorporating a default RMSD of 0.30 Å, only for non-hydrogen atoms, into the filtered database to reduce the energy using the OPLS3 force field. After optimization, shape screening was performed using the crystal structure of the ligand osimertinib as a template. We utilized various pharmacophore types in conjunction with a volume-scoring algorithm to accurately assess the drug. The compounds were evaluated based on their shape similarity scores. The assessment was based on pharmacophore characteristics (Rants’o et al., 2022; Jaundoo et al., 2018; Alturki et al., 2025a; Hussein, 2023; Alturki, 2024).

### 2.6 Validation of molecular docking

Molecular docking protocols were used to verify the precision of Maestro Glide in predicting the ligand docking configurations for the protein under examination (Hollingsworth and Dror, 2018). Library screening criteria were employed to redock the cognate ligands into the EGFR C797S receptor. The ligand osimertinib was isolated from its natural complex (PDB: 6LUD) and docked into all three receptor grids using the Glide Extra Precision (XP) docking module within Schrödinger Maestro. The protein structure was pre-processed using the Protein Preparation Wizard. And receptor grids were created surrounding the co-crystallized ligands. The ligand was prepared using LigPrep at pH 7.4, and flexible ligand docking was enabled (Al Khzem et al., 2025; Berendsen et al., 1981; Bowers et al., 2006; Jorgensenet al., 1996; Zhou et al., 2025).

### 2.7 Virtual screening workflow

**FIGURE 1:**
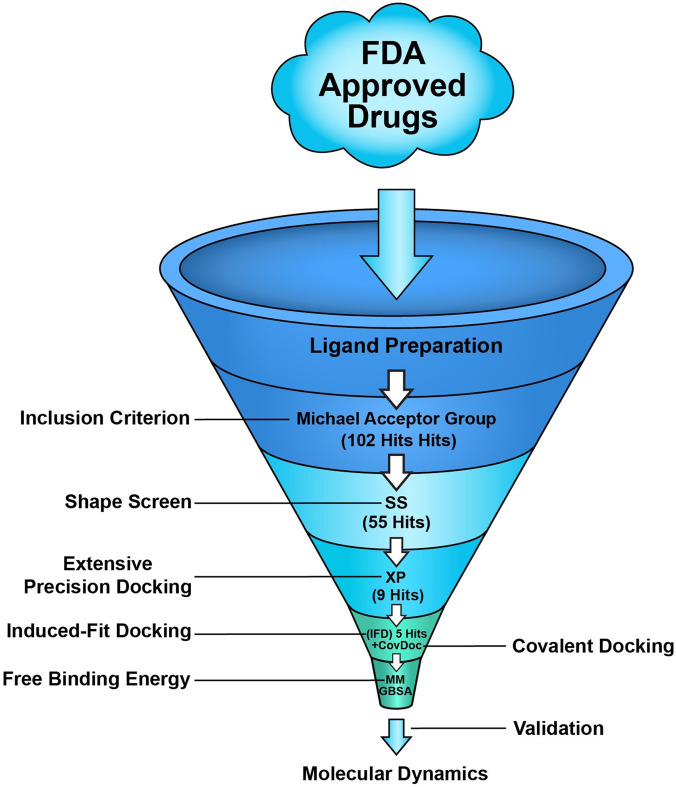
Overview of EGFR C797S receptor *in silico* identification workflow used in this study.

Three separate methodologies were utilized to organize the virtual screening docking workflow: High-Throughput Virtual Screening (HTVS), Standard Precision (SP), and Extra Precision (XP). The parameters were a van der Waals radius scaling factor of 0.80 and a partial charge threshold of 0.15 Å, respectively. Consequently, flexible docking was employed alongside post-docking minimization for each method, yielding three poses per compound and retaining up to 10% of the highest scoring compounds. This filtration criterion is deemed adequate for screening extensive databases (Ikeguchi, 2004). A more stringent hit identification phase was subsequently implemented on the original filter *via* XP docking, MM-GBSA calculations, and 300 ns MD simulations for the final hits (Figure 1) (Alturki et al., 2025b; Guterres and Im, 2020; Kollman et al., 2000; Lippert et al., 2013). No additional filters or constraints were applied during docking. The ligands and hit molecules were ranked using XP scores.

### 2.8 Free binding energy calculations (MM-GBSA)

In molecular mechanics-generalized Born surface area (MM-GBSA) binding free energy calculations, a virtual screening pipeline was used to assess the binding affinity of the highest-ranking candidates using the Prime MM-GBSA module (Genheden and Ryde, 2015) (Figure 1). The following equation was used.

ΔG=GC−GR−GL

where ΔG is the free binding energy, GC is the target/ligand complex energy, GR is the receptor energy, and GL is the ligand. The solvation model was set to VSGB, and the force field was set to OPLS4.

### 2.9 Covalent docking (CovDock)

Covalent docking uses the CovDock module in the Schrödinger Suite (Release 2025-1) to covalently bind the mutant EGFR C797S residue to the selected electrophilic compounds (Rants’o et al., 2022). The reaction type was assigned as a Michael addition, with the ligand’s electrophilic warhead forming a bond with the nucleophilic hydroxyl group of SER-797. The workflow consisted of three steps: (1) pose prediction, where the ligand was initially docked into the binding site with non-covalent constraints to create plausible pre-reaction orientations; (2) covalent bond formation, where the ligand warhead reacted with the designated residue to produce a covalently bound complex; and (3) complex refinement, which involved local side-chain minimization and geometry optimization of the protein-ligand adduct using the OPLS3e force field. The default CovDock parameters were used unless stated otherwise, with flexible ligand sampling turned on and a van der Waals scaling factor of 0.80 applied to the ligand atoms to improve the sampling close to the reactive residue. Docking scores before and after the reaction were examined, and the resulting covalent adducts were visually checked to ensure proper bonding geometry, orientation towards SER-797, and compatibility with the ATP-binding pocket.

### 2.10 Molecular dynamics (MD) simulations

MD simulations were performed for ertapenem, doripenem, norethisterone, norgestrel, oxymetholone, and the co-crystallized ligand osimertinib, which served as a control compound. The Desmond module of the Schrödinger suite (Berendsen et al., 1981; Friesner et al., 2025; Schrödinger Release, 2025, 2025a) was used to conduct the simulations in triplicate. Each protein-ligand complex was solvated using a simple point charge (SPC) water model (Schrödinger Release, 2025, 2025b) in an orthorhombic box with 10 × 10 × 10 Å buffer distances. Next, each system was neutralized by adding counterions, and the ionic strength was adjusted to 0.15 M NaCl. The OPLS5 force field (Alturki et al., 2025c; Jorgensen et al., 1996) was used to run 300 ns MD simulations in triplicate under the NPT ensemble. For these simulations, the temperature was set to 300 K and the pressure to 1 bar. The model system was relaxed before the simulation was performed. The trajectory coordinates were recorded every 300 ps, resulting in 1,000 frames for analysis. The Nose-Hoover chain coupling method with a 1 ps coupling constant was used to control the temperature. For pressure control, the Martyna-Tobias-Klein coupling scheme and a 2 ps coupling constant were used. A 2 fs integration time step was used with the Reference System Propagator Algorithm (RESPA) integrator, and short-range Coulombic interactions were computed with a 9 Å cutoff. The MD simulation data were analyzed using the Simulation Interaction Diagram tool in Desmond (Berendsen et al., 1981; Friesner et al., 2025; Schrödinger Release, 2025, 2025a). RMSD values were measured to assess the stability of the protein-ligand complexes.

## 3 Results and discussion

### 3.1 Database preparation for virtual screening

#### 3.1.1 Shape screening

The cognate ligand was evaluated against a curated database of 1,650 FDA-approved medicines using the shape-screening functionality of the Schrödinger program. A threshold for form similarity indices of ≥0.3 was employed to enhance the shape screening outcomes (Patil et al., 2025).

### 3.2 Virtual screening

**FIGURE 2:**
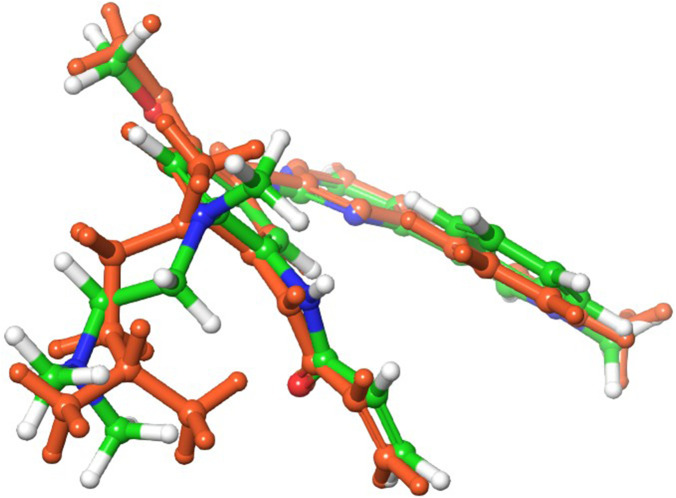
Comparison of binding poses of the co-crystal ligand (green sticks, carbon atom color) and the redocked ligand (orange sticks, carbon atom color), with an RMSD of 0.57 Å.

Docking was first validated by redocking the cognate ligand (osimertinib) into its respective target (EGFR C797S). An RMSD value of less than 2 Å is commonly considered the optimal criterion for ensuring accurate docking (Bonczek et al., 2018; Rajagopal et al., 2017). The results revealed an accurate glide prediction, with an RMSD of 0.57 Å (Figure 2).

**FIGURE 3:**
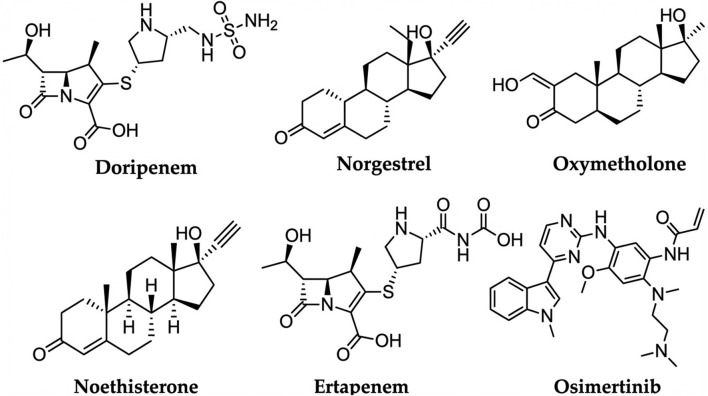
2D structures of the five promising hits and Osimertinib.

The generated databases underwent three phases of virtual screening using Glide: high-throughput virtual screening (HTVS), standard precision (SP), and extra precision (XP) docking. The XP docking study yielded five hits: doripenem, norgestrel, oxymetholone, norethisterone, and ertapenem (Figure 2). The binding affinities of the five hits were compared to the reference, osimertinib, and evaluated against EGFR C797S (PDB code: 6LUD). The inhibitory profiles of the five hits were originally examined *via* docking at the binding site of the specified target, and their binding patterns, target interactions, and binding affinities were analyzed and compared with those of the reference compound, osimertinib (Figure 3).

**FIGURE 4:**
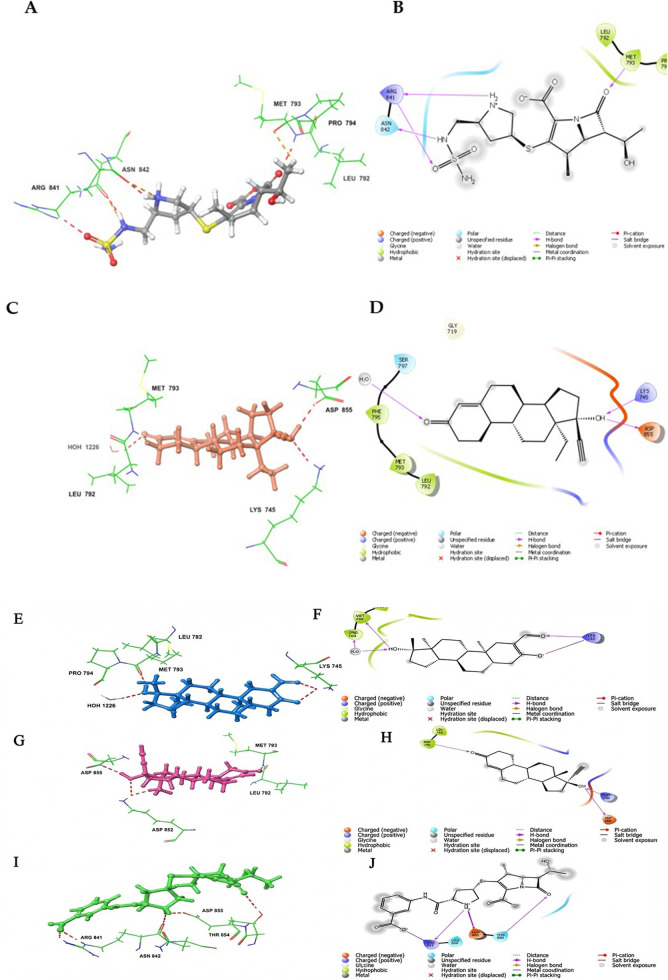
**(A,B)** 3D and 2D representation of the binding interactions between doripenem and EGFR C797S (PDB ID: 6LUD). Ligand atoms are represented as sticks (with carbon atoms depicted in grey), whereas the essential residues are illustrated as sticks (with carbon atoms displayed in green). **(C,D)** Three-dimensional and two-dimensional representations of the binding interactions between norgestrel and EGFR C797S (PDB ID: 6LUD). Ligand atoms are represented as sticks, with carbon atoms depicted in a pale orange hue. **(E,F)** Three-dimensional and two-dimensional representations of the binding interactions between oxymetholone and EGFR C797S (PDB ID: 6LUD). Ligand atoms are represented as sticks, with carbon atoms depicted in azure. **(G,H)** Three-dimensional and two-dimensional representations of the binding interactions between norethisterone and EGFR C797S (PDB ID: 6LUD). Ligand atoms are shown as sticks, with carbon atoms depicted in a muted salmon hue. **(I,J)** Three-dimensional and two-dimensional representations of the binding interactions between ertapenem and EGFR C797S (PDB ID: 6LUD). Ligand atoms are represented as sticks, with carbon atoms depicted in a muted green hue.

The docking results presented in Figure 4 and Table 1 provide a rich foundation for medicinal chemistry analysis, highlighting both the energetic and structural factors that support the repurposing potential of several FDA-approved drugs against the resistant EGFR C797S mutation (PDB ID: 6LUD). The five top-ranking hits (doripenem, norgestrel, oxymetholone, norethisterone, and ertapenem) displayed extra-precision (XP) docking scores ranging from −7.69 kcal/mol for doripenem to −5.08 kcal/mol for ertapenem, reflecting favorable binding energies compared to the co-crystallized control ligand osimertinib (XP score = −10.86 kcal/mol). This trend is reinforced by the MM-GBSA ΔG_bind values, which range from −73.77 kcal/mol (norethisterone) to −44.54 kcal/mol (ertapenem). Although these free energy estimates were less negative than those of osimertinib (−93.41 kcal/mol), they still indicated robust ligand–receptor interactions and a strong thermodynamic basis for complex formation. Although the MM-GBSA showed a wide range among hits (−44.54 to −73.77), the consistent improvement in the binding energies after performing the MM-GBSA calculations indicated favorable binding. A more significant correlation was observed between the MM-GBSA scores of the hits and the experimental reference compound, which provides a more valid criterion for prioritizing hits for experimental validation than the docking scores. From a structural standpoint, the docking poses revealed consistent engagement of key amino acid residues that line the ATP-binding pocket and the gatekeeper region. For example, doripenem established hydrogen-bond interactions with MET-793, MET-794, ARG-841, and ASN-842 (Figures 4A,B). Whereas norgestrel maintained contact with MET-793, H_2_O-1226, and ASP-855 (Figures 4C,D). Oxymetholone and norethisterone were also anchored to MET-793, with oxymetholone also forming links to LYS-745 and water molecules (Figures 4E,F) and norethisterone to LYS-745 and ASP-855 (Figures 4G,H). Ertapenem showed notable ionic interactions with ARG-841 and THR-845, as well as a key hydrogen bond with ASP-855 (Figures 4. I,J). Collectively, these residues flank the hinge region adjacent to the gatekeeper methionine, a site whose steric environment is dramatically altered by threonine-to-methionine (T790M) and cysteine-to-serine substitutions at position 797. Occupying this region with stable hydrogen-bond donors and acceptors is crucial for outcompeting ATP and suppressing downstream signaling cascades. Covalent docking simulations further indicated that all five hits could approach SER-797, a critical mutant residue, in geometries conducive to potential covalent bond formation (Supplementary File S1). Although serine is less nucleophilic than cysteine, its nucleophilicity can be markedly enhanced by the surrounding microenvironment, as exemplified by the activated serines in esterases that perform nucleophilic attacks on ester and amide substrates. Critically, the proximity of anionic ASP800 to SER-797 positions it to deprotonate the serine hydroxyl group, thereby substantially increasing SER-797 nucleophilicity and enabling effective covalent engagement, which is analogous to the mechanisms observed in covalent kinase inhibition pathways. However, the electrophilic motifs present in some of these molecules may still support their irreversible binding. In particular, the β-lactam antibiotics doripenem and ertapenem possess strained four-membered lactam rings that are intrinsically electrophilic, making them attractive warhead candidates for covalent capture by the enzyme. Their heteroatom-rich scaffolds also account for the dense hydrogen-bonding networks. Conversely, the three steroidal compounds, norgestrel, oxymetholone, and norethisterone, present rigid tetracyclic cores that provide excellent shape complementarity to the hydrophobic cleft, and their hydroxyl and carbonyl substituents serve as hydrogen bond donors/acceptors. These steroid scaffolds can be further optimized by appending a Michael acceptor group or a similar electrophilic group at peripheral positions projecting toward SER-797, thereby converting them into covalent inhibitors without disrupting the sterane framework.

**TABLE 1:** Structure, docking scores, MM-GBSA, and shape similarity of the five hits with EGFR C797S binding pocket (PDB ID: 6LUD).

Hit no.	Name	XP score	Induced-fit docking (IFD) (Flexible)	Covalent docking (CovDock)	MM-GBSA dG bind	Shape similarity^a^	Ionic interactions	H-bond interactions	Use
1	Doripenem	−7.69	−9.128	−6.258	−50.41	0.254	​	MET-793, MET-794, ARG-841, ASN-842	Antibiotic
2	Norgestrel	−6.234	−8.490	−3.713	−61.34	0.230	​	MET-793, H2O-1226, ASP-855	Contraceptive
3	Oxymetholone	−5.569	−7.923	−3.140	−57.39	0.256	​	LYS-745, MET-793, H2O-1226	Anemia caused by a deficiency in red cell production
4	Norethisterone	−5.206	−8.309	−4.806	−73.77	0.194	​	LYS-745, MET-793, ASP-855	Abnormal uterine bleeding
5	Ertapenem	−5.084	−9.111	−5.611	−44.54	0.255	ASP-855	ARG-841, THR-845	Antibiotic
Control	Osimertinib	−10.859	NA	NA	−93.41	1.000	ASP-800, GLU-804	MET-793, SER-797	​

^a^ Shape similarity of the hits and the control. Similarity ranges: 0–1.

The shape similarity scores for osimertinib suggest that these hits adopt non-canonical binding modes rather than simply mimicking the co-crystallized ligand. From a medicinal chemistry perspective, such structural diversity is advantageous because it may help circumvent the resistance mechanisms associated with osimertinib-like scaffolds. Despite the expected off-target effects from their diverse therapeutic origins—antibiotics, contraceptives, and anemia treatments—these FDA-approved hits harbor substantial potential for clinical development against EGFRC797S-mutant NSCLC, a serious disease with poor prognosis and limited alternatives following osimertinib resistance (occurring in 10%–40% of cases *via* the less-nucleophilic C797S mutation that abrogates covalent binding), where current therapies, such as third-generation TKIs, plateau at approximately 2-year Progression-Free Survival and lack approved options for triple mutants. Conversely, their chemical scaffolds represent promising starting points for targeted optimization, enabling the design of potent and selective new-generation EGFR inhibitors tailored to C797S-mutant NSCLC. In summary, the combined docking metrics, residue-level interactions, covalent-binding potential, and chemical diversity described in Table 1 provide a compelling foundation for advancing these repurposed candidates toward clinical translation and lead optimization to overcome the C797S resistance mutation.

### 3.3 Molecular dynamics (MD) simulations

In a previous study, the inhibitor potency against Del19/T790M/C797S triple-mutated EGFR was attributed to multiple non-covalent interactions between the inhibitor and the binding site, including the mutant residue T790M (Kumar and Zhang, 2018). These findings were based on the differences in potency and binding between osimertinib and CH7233163, as observed in the experimentally determined protein-inhibitor complexes (Kumar and Zhang, 2018). Compared to osimertinib, which can be ineffective against Del19/T790M/C797S triple-mutated EGFR, CH7233163 is more potent, binds deeper in the ATP-binding pocket, and interacts uniquely with the mutant gatekeeper residue T790M (Kumar and Zhang, 2018). Inhibitor binding in an αC-helix-in conformation is required for effective inhibition of the Del19/T790M/C797S triple-mutated EGFR (Kumar and Zhang, 2018). CH7233163 binds to the L858R/T790M/C797S triple-mutated EGFR in an αC-helix-in conformation (Kumar and Zhang, 2018). This binding mode has been previously linked to potent inhibition of the Del19/T790M/C797S variant, which may be unable to adopt an αC-helix-out conformation (Kashima et al., 2020).

Molecular dynamics (MD) simulations were performed on protein-ligand complexes of the five hits (ertapenem, doripenem, norethisterone, norgestrel, and oxymetholone) and the co-crystallized ligand (osimertinib, the control compound) to evaluate their docking poses and assess their stabilities and binding behavior in a dynamic environment. Some of these hits have functional groups capable of covalent binding. Classical MD simulations typically do not capture covalent bond formation. Nevertheless, they offer valuable insights into ligand-binding stability and interaction persistence, providing a structural context that can support covalent or non-covalent binding modes.

**FIGURE 5:**
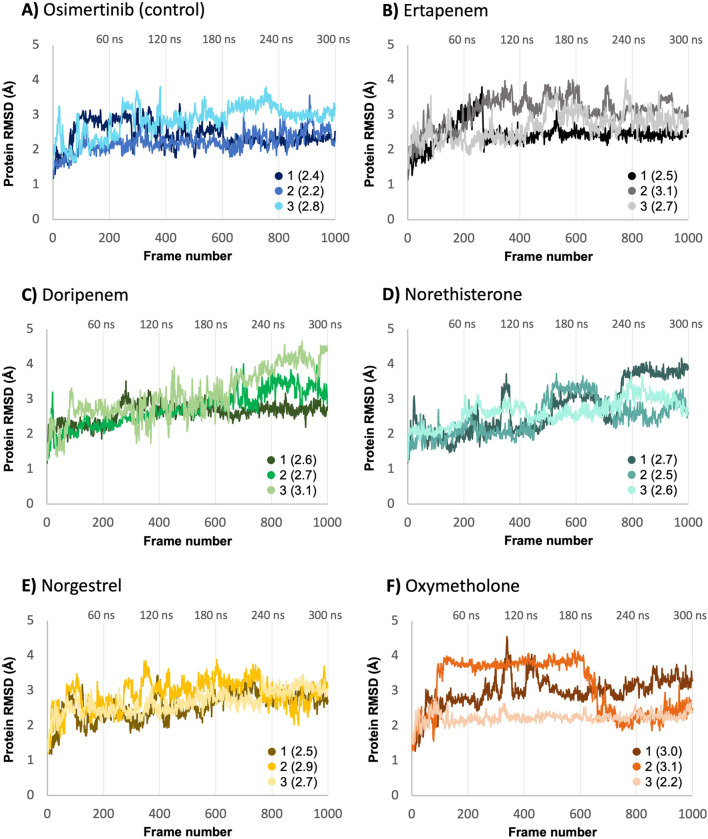
Protein Cα atom RMSD analysis from each protein-ligand complex **(A–F)** based on triplicate MD runs (shown as 1, 2, and 3). Average RMSD values are reported in parentheses.

RMSD analysis of Cα atoms was used to assess the stability of each protein-ligand complex (Figure 5). No significant fluctuations were observed for protein Cα atoms in the complexes of ertapenem, norethisterone, norgestrel, and the control (osimertinib). These minor fluctuations fall within an acceptable range of 1–3 Å, indicating that the protein in each complex was stable and equilibrated throughout the simulation across all three replicates. In the cases of the doripenem and oxymetholone complexes, some fluctuations in the protein Cα atoms were slightly higher than 3 Å, but remained below 3.5 Å. Interestingly, these larger fluctuations were observed only in one of the replicate MD runs for each of the doripenem and oxymetholone complexes, replicate 3 for doripenem and replicate 1 for oxymetholone. In both cases, the protein stabilized within the first 30 ns. Smaller fluctuations were observed in the other two replicates, which fall within the acceptable range of 1–3 Å. These findings indicate overall protein stability. Overall, no significant structural deviations were observed for the protein in all complexes. Average RMSD values for protein Cα atoms ranged from 2.2 to 3.1 Å, all being close to average RMSD values for the protein in complex with the control (osimertinib) that ranged between 2.2 and 2.8 Å.

**FIGURE 6:**
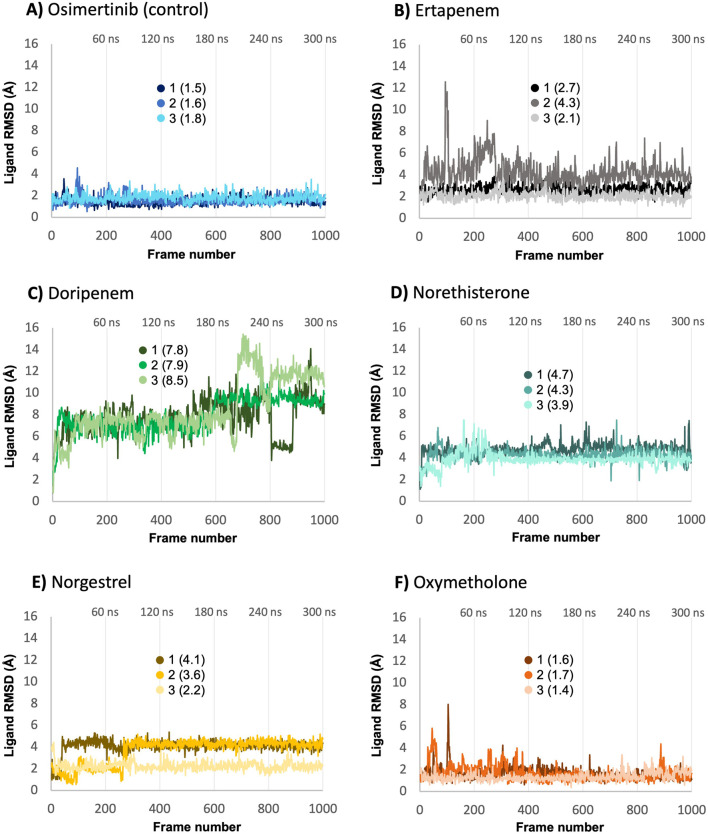
Ligand heavy-atom RMSD analysis from each protein-ligand complex **(A–F)** based on triplicate MD runs (shown as 1, 2, and 3). Average RMSD values are reported in parentheses.

The RMSD analysis of the heavy atoms of the ligands was performed to assess the stability of each ligand relative to its corresponding protein within the complex (Figure 6). Except for doripenem, the average ligand heavy-atom RMSD values for all compounds across replicates were very close to their corresponding average Cα atom values, indicating stable binding of these compounds within their protein-binding sites. In contrast, doripenem had higher average ligand-heavy-atom RMSD values (7.8, 7.9, and 8.5 Å) than the average Cα RMSD values of its protein (2.6, 2.7, and 3.1 Å), indicating unstable binding and primary ligand structural changes within the binding site.

The control compound, osimertinib, had average ligand-heavy atom RMSD values ranging from 1.5 to 1.8 Å. For the five hit compounds, the average RMSD values for the ligand-heavy atoms ranged from 1.4 to 8.5 Å. Notably, oxymetholone had average RMSD values below 2 Å across replicates, indicating that it was stably bound within its binding site, similar to the values and binding behavior of the control. In contrast, doripenem had the highest average ligand RMSD values, ranging from 7.8 to 8.5 Å, indicating the least stable binding. This is consistent with the earlier observation of large differences between the ligand and protein RMSD values.

**FIGURE 7:**
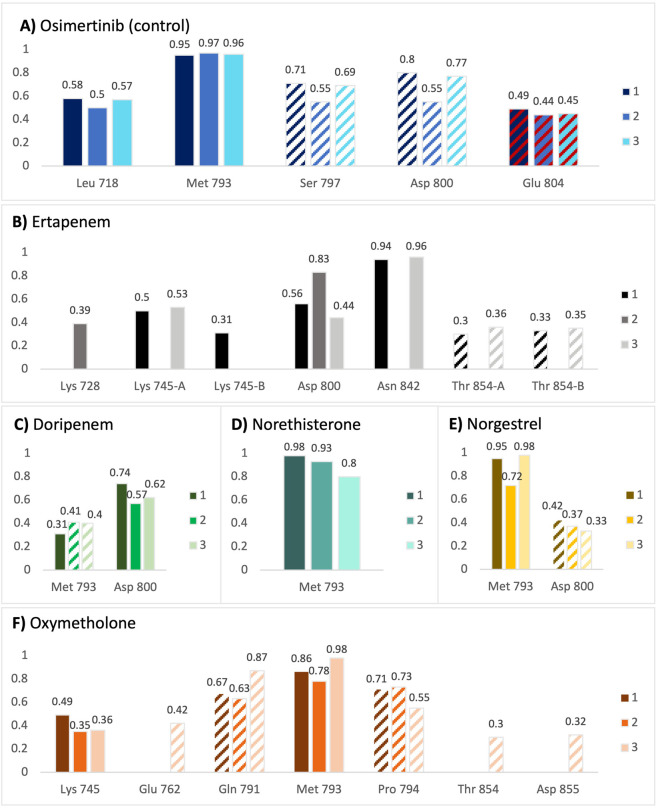
Fractional interaction occupancies for each of the compounds **(A–F)** with triple-mutant EGFR residues across triplicate MD simulations (shown as 1, 2, and 3). Solid-colored bars represent hydrogen bonds, diagonally striped bars on a white background represent water bridges, and diagonally striped bars on a red background represent salt bridges.

**FIGURE 8:**
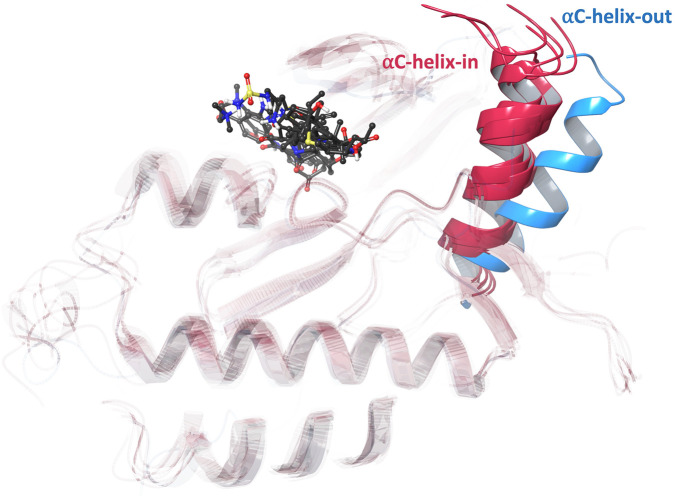
Superposition of representative MD structures (replicate 3) for each compound (proteins shown in red ribbons; ligands in grey ball-and-stick representation) with the allosterically bound mutant EGFR structure in an αC-helix-out conformation [blue ribbon; PDB ID: 5D41 (Bonczek et al., 2018)]. The allosteric inhibitor is not shown for clarity.

Ligand-protein binding was assessed by visual inspection and analysis of the interaction persistence. Interactions were considered strong and stabilizing if they persisted for more than 50% of the simulation time. Detailed interaction diagrams are shown in the supplementary figures. In all replicates, osimertinib was strongly held in the binding site *via* strong hydrogen bonds with LEU-718 and MET-793, as well as stable water bridges to ASP-800 and the mutant residue SER-797 (C797S) (Figure 7A). The compound also formed a salt bridge with GLU-804, which remained for 44%–49% of the simulation time period. Osimertinib exhibited minimal fluctuations within the binding site, supporting the observed low average heavy-atom RMSD values. Figure 6. Fractional interaction occupancies for each of the compounds (A–F) with triple-mutant EGFR residues across triplicate MD simulations (shown as 1, 2, and 3). Solid-colored bars represent hydrogen bonds, diagonally striped bars on a white background represent water bridges, and diagonally striped bars on a red background represent salt bridges. In replicates 1 and 3, ertapenem appeared stable, as indicated by the low variation in heavy-atom RMSD values. Ertapenem was held in place *via* strong and stable hydrogen bonds with ASN-842 and LYS-745, and in replicate 1, with ASP-800 (Figure 7B), with minor structural variation. In replicate 2, the pyrrolidine ring in ertapenem formed a strong hydrogen bond with ASP-800; however, the S-linked carbapenem ring substituent did not bind strongly to any of the amino acids, explaining its movement within the binding site and the corresponding RMSD value fluctuations. Overall, across the two replicates, ertapenem bound strongly and stably to the binding site. In all three replicates, the pyrrolidine ring in the other β-lactam antibiotic, doripenem, also participated in strong hydrogen bonding with ASP-800; however, the rest of the compound did not stably bind to any residue (Figure 7C), leading to large structural variations and the observed large ligand heavy-atom RMSD fluctuations. Given this apparent binding instability, doripenem is unlikely to maintain a productive binding mode and is therefore not considered a strong or promising candidate, despite its good initial docking score. The three steroids, norgestrel, norethisterone, and oxymetholone (Figures 7D–F), formed strong hydrogen bonds with the MET-793 backbone through their carbonyl groups. Notably, both norgestrel and norethisterone changed their orientations at the beginning of the simulation to orient their carbonyl groups in optimal positions for forming a persistent hydrogen bond with the MET-793 backbone, a behavior observed in all three replicates. In contrast, oxymetholone did not change its orientation, as it was held in place by additional persistent water bridges to GLN-791 and PRO-794, maintaining an optimal position for carbonyl hydrogen bonding with MET-793. The three steroids lack functional groups that are sufficiently electrophilic to react with SER-797. However, the stable binding of oxymetholone suggests the potential for non-covalent binding or further optimization through the introduction of electrophilic warheads. Similar to the control compound, osimertinib, all compounds occupied the ATP-binding pocket and did not form strong interactions with the gatekeeper residue MET-790. This binding behavior is distinct from that observed for the potent CH7233163. Notably, osimertinib was the only compound that formed a stable hydrogen bond with the mutant residue SER-797 (C797S). All compounds from the MD simulations adopted the C-helix-in conformation, as expected for potent inhibitors of triple-mutated EGFR (Figure 8). Overall, based on the RMSD analysis and interaction profiles, ertapenem and oxymetholone exhibited the most stable binding to mutant EGFR, with minimal structural deviations throughout the simulation. These two compounds formed multiple strong hydrogen bonds throughout most of the simulation time across all replicates, similar to the control compound, osimertinib, and consistent with previously reported observations for CH7233163 binding (Kumar and Zhang, 2018). Although the docking and MM-GBSA scores of the selected compounds were lower than those of osimertinib, this was expected because osimertinib was optimized for wild-type EGFR. Significantly, the C797S mutation disrupts the irreversible binding of osimertinib; therefore, the predicted affinity alone is not sufficient to determine the efficacy against the resistant variant. The stable MD interactions observed for ertapenem and oxymetholone highlight their potential as starting points for targeting EGFR C797S through noncovalent mechanisms.

### 3.4 Comparison with previous studies targeting EGFR C797S

Previous research efforts to overcome the EGFR C797S resistance obstacle have focused on developing fourth-generation tyrosine kinase inhibitors. CH7233163 was discovered to function as a potent EGFR inhibitor by stabilizing the kinase in an αC-helix-in state and binding to the hinge region without creating T790M gatekeeper mutation obstructive effects (Kashima et al., 2020). Crystallographic and biochemical methods demonstrated that CH7233163 enters the ATP pocket more deeply than osimertinib while establishing novel binding patterns with MET-790, which results in its superior effectiveness against the triple mutant. In this study, molecular dynamics simulations indicated that all shortlisted repurposed compounds, including ertapenem and oxymetholone, adopted αC-helix-in conformations of the EGFR kinase domain, aligning with the binding mode previously linked to potent inhibition of C797S-containing mutants. However, unlike CH7233163, none of the repurposed compounds formed stable interactions with the gatekeeper residue MET-790. This finding reflects the behavior of osimertinib against C797S and provides a mechanistic rationale for considering the identified compounds as initial scaffolds rather than optimized inhibitors. Despite this limitation, ertapenem and oxymetholone maintained stable hinge-region hydrogen bonds to MET-793 and consistent interaction networks over 300-ns triplicate simulations, comparable to the reference ligand, underscoring their potential for further medicinal-chemistry optimization. Recent computational studies have often used docking and molecular dynamics approaches on EGFR-mutant systems, typically focusing on newly designed quinazoline or pyrimidine scaffolds and assessing binding through short (50–100 ns) single-trajectory simulations (Sakuma et al., 2012). In contrast, this study extends previous methodologies by employing a hierarchical FDA-drug repurposing campaign combined with electrophilic filtering, covalent-docking protocols, and long-timescale triplicate molecular dynamics simulations totaling 900 ns per compound. This more rigorous dynamic evaluation allowed for distinguishing between seemingly favorable docked poses, such as that of doripenem, and ligands that remained stably anchored throughout the simulations, thereby reducing false-positive prioritization. Computational campaigns focused on repurposing against resistant kinases have previously emphasized the benefit of identifying chemically diverse scaffolds distinct from approved TKIs, particularly to bypass cross-resistance mechanisms (Qureshi et al., 2021; Qureshi et al., 2022). In line with this concept, our top-ranked hits belong to two unexpected chemical classes—β-lactam antibiotics and steroidal agents—rather than traditional EGFR-inhibitor chemotypes. The identification of ertapenem and oxymetholone thus broadens the chemical space currently explored for C797S targeting and suggests new directions for scaffold-hopping and warhead installation strategies aimed at restoring irreversible or high-affinity reversible binding. Overall, while previous studies have reported optimized experimental inhibitors like CH7233163 with sub-nanomolar potency, this investigation differs in both scope and intent: rather than proposing immediate therapeutic candidates, we establish a computational repurposing framework that nominates clinically approved molecules as viable starting points for rapid experimental validation and lead optimization. The agreement in αC-helix-in stabilization and hinge-binding modes with earlier structural studies supports the biological plausibility of the identified hits, while the lack of strong MET-790 engagement highlights the need for rational medicinal-chemistry refinement in future work.

## 4 Conclusion

This study developed a complete drug repurposing method that used multiple computational stages to solve the main medical problem that third-generation EGFR-tyrosine kinase inhibitors face in non-small cell lung cancer treatment because of the C797S mutation. Multiple techniques were implemented, including electrophilic filtering, shape-based screening, hierarchical molecular docking, MM-GBSA binding free-energy calculations, covalent-docking protocols, and long-timescale triplicate molecular-dynamics simulations, to create a new process for identifying FDA-approved drugs that could treat resistant EGFR. Among the five hits, ertapenem and oxymetholone were the most promising. The agents maintained continuous binding to the ATP-binding pocket during the 300-ns simulation period while establishing permanent hydrogen bonds in the hinge region with MET-793 and displayed interaction behavior similar to that of the reference inhibitor osimertinib during the same dynamic conditions. Doripenem exhibited sustained ligand mobility and transient bonding, demonstrating that molecular dynamics simulations serve as an essential post-docking filter rather than merely a method to verify results. Although several candidates possess electrophilic functionalities capable of approaching SER-797, our findings indicate that stable non-covalent engagement of the hinge and gatekeeper-adjacent regions is likely the dominant determinant of binding for the identified repurposed scaffolds. The chemical diversity of the top hits, spanning β-lactam antibiotics and steroidal frameworks, highlights previously unexplored regions of chemical space for EGFR C797S targeting and provides tractable starting points for scaffold-hopping, warhead installation, and structure-guided optimization.

Importantly, this investigation is inherently predictive and does not substitute experimental validation. Biochemical kinase-inhibition assays, cellular evaluation in EGFR-mutant NSCLC models, and pharmacokinetic profiling are required to determine whether these compounds or their optimized analogs can achieve therapeutically relevant activity. Nevertheless, the present study establishes a transferable computational framework for rapid repurposing campaigns against kinase resistance mutations and nominates ertapenem- and oxymetholone-derived chemotypes as rational leads for future medicinal chemistry programs aimed at overcoming osimertinib resistance in lung cancer.

## Data Availability

The original contributions presented in the study are included in the article/Supplementary Material, further inquiries can be directed to the corresponding author.
