# Supplementary material for: A multi-stage computational pipeline for repurposing FDA-approved drugs: application to EGFR C797S–mutant NSCLC
Source: Front Chem. 2026 Mar 31;14:1753911. doi: 10.3389/fchem.2026.1753911 (PMC13077434; doi:10.3389/fchem.2026.1753911)
Supplement: Supplementary file 1 [file DataSheet1.pdf]

Supplementary Material

1 Supplementary Data

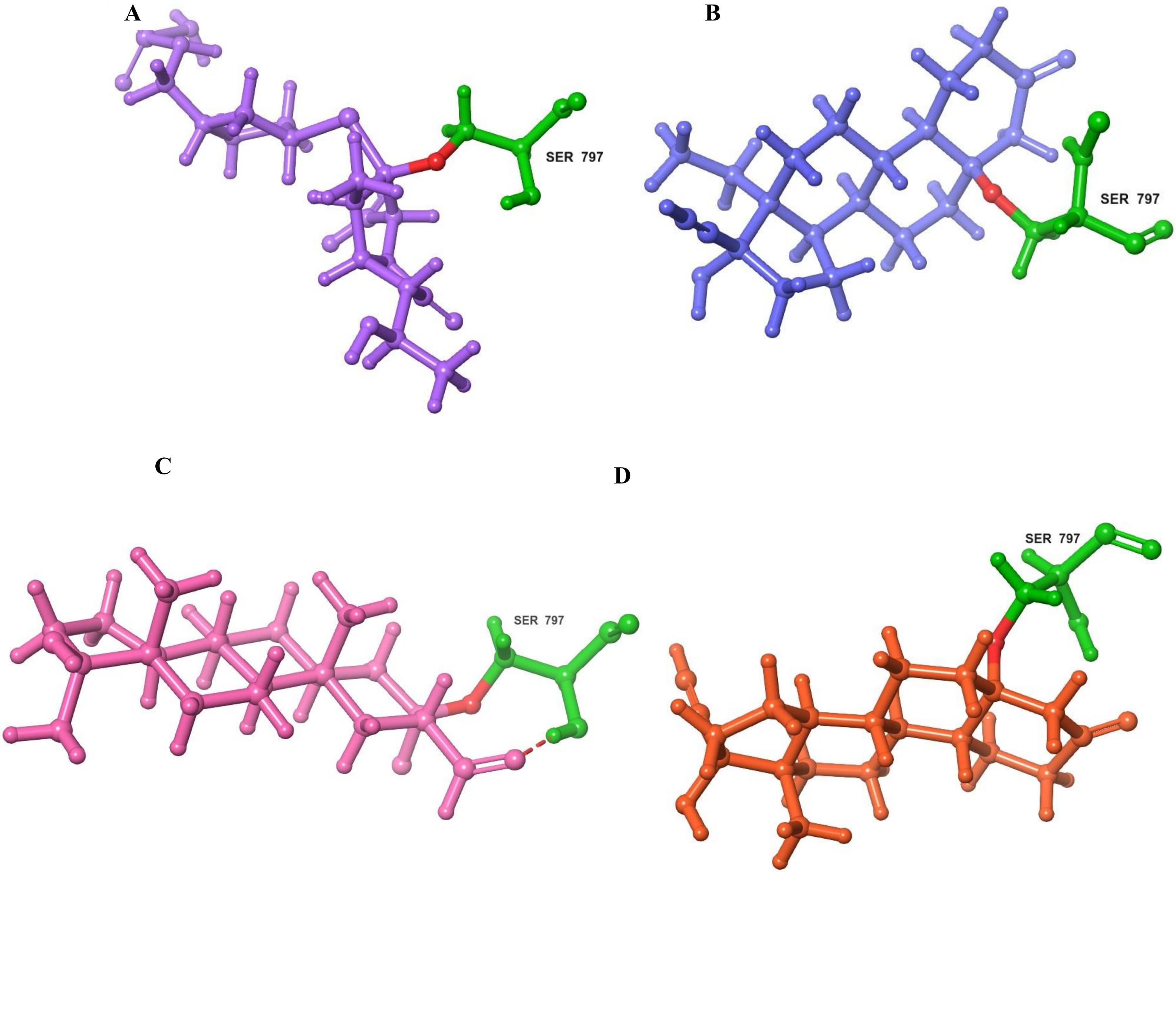

E

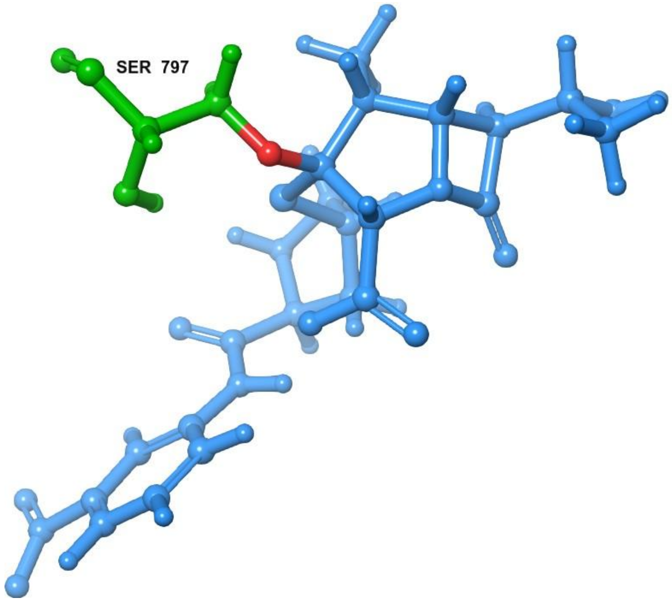

Supplementary Figure 1: A. Doripenem, B. Norgestrel, C. Oxymetholone, D. Norethisterone, and E. Ertapenem. Covalent docking results show the predicted binding modes of the tested compounds within the EGFR active site, highlighting covalent interactions with Ser797. The ligand structures are shown in colored stick representation (each compound in a distinct color), while Ser797 is depicted in green. The covalent bond formed between the ligand's electrophilic warhead and the oxygen atom of Ser797 is represented in red.
